# Hodgkin Lymphoma, Tuberculosis, and Atypical Radiologic Image

**DOI:** 10.4274/tjh.galenos.2019.2019.0291

**Published:** 2019-11-18

**Authors:** Sora Yasri, Viroj Wiwanitkit

**Affiliations:** 1 KMT Primary Care Center, Bangkok, Thailand; 2 Joseph Ayobabalola University, Ikeji-Arakeji, Nigeria

**Keywords:** Hodgkin Lymphoma, Tuberculosis, Radiology

## To the Editor,

We read the report by Büyükşimşek et al., [1] “Atypical Radiologic Image Characterized by Cavitary Lung Lesions in a Case of Hodgkin Lymphoma” (HL), with great interest. Büyükşimşek et al. [1] reported on a case of HL presenting with abnormal lung radiologic imaging and mentioned that “Disseminated cavitary lesions mimicking tuberculosis or other opportunistic infections in a case of HL is interesting and differential diagnosis is very important”. We would like to share our ideas regarding this observation. Indeed, lung involvement due to lymphoma is possible. Nevertheless, the concurrence between HL and tuberculosis is detectable. In endemic areas of tuberculosis, such as Southeast Asia, tuberculosis screening is routinely done for any cancerous patients, including those with HL. Pathophysiologically, a common pathway that can result in increased risk for tuberculosis among patients with HL is the alteration of the antioxidative system. The depletion of glutathione (GSH) due to HL [2] can increase the risk for tuberculosis since GSH plays an important role in defending against mycobacterial pathogens [3]. Considering the present report by Büyükşimşek et al., [1] there is an interesting question of whether the present case of HL had a concurrent tuberculosis infection or not. Büyükşimşek et al. [1] used the QuantiFERON test for exclusion of tuberculosis. In a recent report, the sensitivity and specificity of the QuantiFERON test were found to be poor [4]. In cases with underlying vitamin B12 deficiency, false negative results by QuantiFERON are possible [5]. In a recent report, vitamin B12 deficiency was observable in 0.54% of patients with HL and anemia [6].
