# Supplementary material for: Hodgkin Lymphoma, Tuberculosis, and Atypical Radiologic Image
Source: Turk J Haematol. 2019 Nov 18;36(4):301–2. doi: 10.4274/tjh.galenos.2019.2019.0291 (PMC6863025; doi:10.4274/tjh.galenos.2019.2019.0291)
Supplement: Related texts

# Reply from the Authors

## To the Editor,

We thank Drs. Yasri and Wiwanitkit for their interest and for sharing their thoughts on our case report. We agree with them about the co-occurrence of tuberculosis and lymphoma, especially in endemic areas. Additionally, it is very well known that infections with *Mycobacterium tuberculosis* and other intracellular microorganisms are common in cases of Hodgkin lymphoma (HL) due to underlying T-cell defects [1,2]. On the other hand, clinical symptoms and signs including fever, night sweats, and weight loss are very common in tuberculosis and in HL, and sometimes it may be very difficult to differentiate HL and/or accompanying tuberculosis in a case of HL. For this reason, as we discussed before, tuberculosis was the first diagnosis in our case when the patient presented with fever and night sweats. To differentiate and to exclude tuberculosis, we tried different technologies, including culture for tuberculosis and follow-up radiologic imaging, and also clinical signs and symptoms. Of course QuantiFERON was not the only applied test in our case, but due to the journal’s space limitations we could not mention the other tests: culture for tuberculosis was reported as negative and the patient responded very well to anti-lymphoma therapy only.

## References

[1] Roux M, Schraven B, Roux A, Gamm H, Mertelsmann R, Meuer S (1991). Natural inhibitors of T cell activation in Hodgkin’s disease. Blood.

[2] Melero M, Gennaro O, Domínguez C, Sánchez Avalos JC (1992). Tuberculosis in patients with lymphomas. Medicina (B Aires).
